# Progress in Molecular Plant Science (2023–2025)

**DOI:** 10.3390/cimb48010043

**Published:** 2025-12-27

**Authors:** Zhaohui Chu

**Affiliations:** State Key Laboratory of Hybrid Rice, Hubei Provincial Research Center for Basic Biological Sciences, College of Life Sciences, Wuhan University, Wuhan 430072, China; zchu77@whu.edu.cn

## 1. Introduction

The past three years (2023–2025) have witnessed substantial progress in molecular plant science, driven by technological innovations and deepened mechanistic insights. Key advancements span disease resistance breeding, understanding of biotic and abiotic stress responses, plant–microbe/insect interactions, genome editing, and multi-omics applications. This editorial highlights major developments in these areas, illustrating their collective impact on fundamental science and agricultural improvement.

## 2. Advances in Wheat Research and Gene Cloning

Breeding and utilizing resistance genes are the most economical and effective strategies for controlling various plant diseases. Over the past three years, several fungal disease resistance genes have been cloned in wheat [1,2,3]. Additionally, a rapid cloning system capable of isolating a new gene within six months has been developed [4]. Marked by the fast-cloning platform established in 2025, high-throughput and efficient gene cloning is now feasible, dramatically accelerating gene discovery from the large and complex wheat genome. Notable examples include the following:

A novel immune mechanism has been identified, revealing that the atypical NLR protein WTN1 collaborates with the tandem kinase WTK3 to recognize pathogen effectors and confer broad-spectrum fungal resistance [1]. Additionally, the broad-spectrum leaf rust resistance gene *Lr47* from *Aegilops speltoides* has been cloned, and short-segment translocation lines have been developed for breeding applications [2]. Furthermore, the powdery mildew resistance gene *Pm57* from *Aegilops searsii*, which encodes a novel tandem kinase protein and provides immunity to all known wheat powdery mildew isolates, has also been successfully cloned [3].

More than 2100 wheat varieties interacting with *Puccinia striiformis,* a causal agent of rust of wheat, have been analyzed. Three loci were identified: *Yr6/Pm5*, *YrKB*, and *TaEDR2-B* [5].

Four distinct tomato resistance genes (*I-1*, *I-2*, *I-3*, and *I-7*) have been identified that confer protection against the fungus *Fusarium oxysporum*. All four immune receptors trigger the accumulation of *PR-5x*, *PR-P2*, and two glucan endo-1,3-β-D-glucosidases [6].

## 3. Plant–Pathogen Interactions

A novel spotted leaf mutant in rice has been shown to confer resistance to both blast and bacterial blight [7]. In wheat, the cytokine DEP2 was found to regulate disease resistance, providing new insights into immune signaling [8]. During bacterial infection, effectors delivered via the type III secretion system often target host immune components, such as receptor-interacting protein kinases, to promote virulence [9,10,11]. *Citrobacter rodentium* further exemplifies how pathogens deploy complex effector networks to subvert host defenses [10].

## 4. Abiotic Stress Tolerance

Although plants cannot escape abiotic stress by moving, they do not remain passive or simply endure adverse conditions. Instead, they actively employ multiple mechanisms to enhance their stress tolerance. Key genes and mechanisms underlying this tolerance have been identified, including the following:

The alkaline tolerance gene *AT1*, an ortholog of rice *GS3*, alleviates oxidative stress by regulating the phosphorylation of aquaporins [12]. Specifically, *AT1*/*GS3* inhibits the phosphorylation of PIP2 aquaporins, which mediate oxidative stress under alkaline conditions. Knockout of the *AT1* gene in sorghum, as well as its homologs in millet, rice, and maize, enhances the plants’ alkaline tolerance, resulting in higher yields, increased biomass, and improved survival rates when grown in alkaline soils during field trials. These findings suggest that genetic modification of *AT1* homologs in crops could improve productivity in saline-alkaline soils, thereby enabling the cultivation of millions of hectares of sodic land.

Soil salinity also severely limits crop growth development, resulting in a reduction in agricultural production worldwide. Salt stress responses are regulated by ABA signaling, which modulates root sodium ion uptake, minimizing cellular damage [13]. The activation of the CDK8 kinase module under salt stress leads to the degradation of the repressor AHL10 and the induction of tolerance genes [14]. A phosphorylation-based mechanism involving CPK3 fine-tunes immunity and growth under osmotic stress [15]. Additionally, CPK3 mediates copper-induced resistance in *Arabidopsis* by regulating the expression of *bHLH107* and *ACS8* [16].

Drought, characterized by water deficiency, is another abiotic stress that limits plant growth, development, and agricultural yield. Integrated multi-omics analyses have identified conserved regulators of drought response at the cell-type level, such as *CIPK23* [17], and have uncovered alternative splicing mechanisms in barley [18], *Arabidopsis* [19], tobacco [20], and legume [21].

Low temperature is also a kind of abiotic that affects plant growth and development. A genome-wide association study was performed in maize and identified multiple genetic loci involved in tolerance to cold [22]. Intriguingly, Li et al., identified a natural variations in *QT12* conferring to thermotolerance for grain quality and yield in rice [23].

## 5. Plant–Rhizosphere Interactions

Rhizosphere microbes regulate key agronomic traits, such as rice tillering [24], and alleviate aluminum toxicity [25]. Under heavy metal stress, a cross-kingdom signaling loop involving root ROS, microbial IAA, and host DNA methylation enhances plant tolerance [26]. A landmark study demonstrated how root-secreted glutamine guides microbial colonization along Casparian strip “gaps,” establishing a spatial settlement pattern for beneficial microbes [19]. RNA sequence is performed in *Medicago truncatula* and observed the induction of TML1 and TML2 in the Nodulation mutants, suggesting an unknown regulation mechanism [27].

## 6. Plant–Insect Interactions

New genes and signaling pathways contributing to insect resistance have been identified in potato [28], cotton [29], poplar [30,31], and rice [32]. Detailed mechanisms involving resistance genes [33] and non-coding RNAs [34] have been elucidated in rice–brown planthopper interactions.

A tissue-specific defense mechanism has been identified in potato plants. When attacked by the potato tuber moth, undamaged young leaves undergo compensatory growth to enhance tolerance, whereas the damaged leaves trigger resistance responses. Both responses are regulated in a tissue-specific manner by the sugar transporter SWEET11 [28].

In cotton, a multi-omics integrative analysis identified two key proteins, GhDP1 and GhAKR13D2. These proteins synergistically regulate the rerouting of terpenoid defense metabolism in green cotton tissues, leading to increased production of the defense-active compound hemigossypolone and its derivative, heliocides [29].

In Populus, a novel breeding strategy involving the pyramiding of multiple desirable traits was successfully implemented using multi-gene expression technology. By combining the gene encoding the key enzyme for reactive oxygen species scavenging, superoxide dismutase (CSD2), with the Bt protein-encoding gene Cry3A and expressing both in poplar, a new transgenic poplar germplasm exhibiting high yield, superior quality, and enhanced insect resistance was developed [30].

## 7. CRISPR and Genome Editing

CRISPR technology continues to facilitate precise trait improvements, such as increased branching in rapeseed [35]. Similar applications have been developed in peanut [36], a CRISPR-Cas12a system optimized for common wheat [37], and the CRISPR method was used for the rapid detection of fungal pathogens [38] and improving grain quality in rice [39] and banana [40].

To optimize plant architecture in rapeseed, CRISPR/Cas9 technology was employed to target the *Bna.BRC1* homologs, generating knockout mutants. Five mutants were produced, exhibiting significantly increased branching compared to wild-type plants [35]. Additionally, herbicide-resistant allotetraploid peanuts were successfully developed using CRISPR/Cas9-mediated cytosine base editing, specifically targeting the *AhALS* gene.

To enhance the efficiency of gene editing in common wheat, biolistic delivery of intron-optimized LbCas12a and its variants into shoot apical meristems was developed [37]. Similarly, a novel and efficient CRISPR/SpRY system was created for the rapid detection of rice fungal pathogens [38]. This CRISPR/SpRY detection platform can identify single-nucleotide mutations in under one hour, with a detection limit as low as 1%. The CRISPR system provides a powerful method, based on rapid gene targeting, for plant breeding programs [41].

## 8. Multi-Omics and Systems Approaches

Integrated multi-omics analyses have been conducted to reveal signaling pathways associated not only with plant responses to biotic stresses [42,43,44] and abiotic stresses [19,45,46,47,48,49,50] but also to beneficial microbes [51].

*Ustilaginoidea virens*-induced rice false smut reduces both the yield and quality of rice, yet little research has been conducted on resistance genes against false smut. The resistance mechanism of IR28 was examined through RNA sequencing, revealing resistance pathways mediated by CNL proteins [42]. In the poplar–*Alternaria alternata* interaction, RNA sequencing and enzyme activity assays were performed to investigate the molecular mechanisms underlying leaf blight defense in poplar [44]. Studies on combined stresses reveal that plant responses to concurrent drought and low nitrogen cannot be extrapolated from data obtained under single-stress conditions [19]. Phosphorus deficiency responses in rice are regulated via the RLI1-OsPUB77-OsBZR3 module, which shapes plant architecture under low phosphorus conditions [45]. Transcriptomics analysis was performed in Suaeda glauca [46] cereals [47], tea plant [48], fruits [49], and cottons [50] under abiotic stresses. Arbuscular mycorrhizal symbiosis (AMS) is widely distributed and benefits plant growth, development, and stress resistance [51]. In tomato, AMS induces transcriptional alterations in thousands of protein-coding genes, leading to the construction of a tomato symbiotic transcriptome database.

## 9. Plant Hormone Signaling

Plant hormone signaling systems—including salicylate (SA), jasmonate (JA), ethylene, abscisic acid (ABA), auxins, cytokinins, gibberellins, and brassinosteroids—play a key role in activating plant immune responses. In peanuts, focusing on JA signaling [52], a total of 64 genes encoding key enzymes involved in JA biosynthesis were identified. These genes were classified into lipoxygenases, allene oxide synthases, allene oxide cyclases, and 12-oxo-phytodienoic acid reductases based on gene structure, conserved motifs, and phylogenetic characteristics. MeJA was found to upregulate the expression of monoterpenoid biosynthesis in *Schizonepeta tenuifolia* [53]. Except for JA, IAA is found to be involved in the growth of Chinese pine [54] and oliver [55], and gibberellins have a role in the bud growth of *Petunia hybrida* [56].

Salicylic acid (SA) is a key hormone involved in plant immunity against pathogens [57,58]. In rice, the DMR6-like gene *OsF3H04g*, which encodes an SA hydroxylase, has been identified as a susceptibility factor, while its homolog *OsS3H* modulates resistance to bacterial leaf streak. In potato, copper ions also activate the *StRVE1* to increase the expression of *StSID2* for accumulation of SA, which is in addition to the rapidly activated synthesis of ethylene, and the key transcription factor StEIN3 directly inhibits the ABA biosynthesis genes *StABA1* and *StNCED1*, resulting in decreased levels of ABA [59].

## 10. Recovery and Growth Regulation

As climate change progresses, high temperatures are becoming more frequent, limiting agricultural production. The mechanism of temperature sensitivity at the *Sa* locus in sterile *xian*/*indica*–*geng*/*japonica* hybrid rice has been elucidated, revealing the temperature-sensitive system composed of three genes [60]. Genes *LHCB4.1* and *LHCB5* were identified and validated as suitable qRT-PCR reference genes for *Arabidopsis* responses to high-temperature stress [61].

Nitrogen fertilization promotes wheat recovery after drought by nitrate-mediated inhibition of *TaSnRK2.10*, which activates *TaNLP7* and stimulates regrowth [62]. Twelve rice varieties with significant differences in heat tolerance were studied to explore the characteristics and regulatory mechanisms of the effect of ABA-mediated ROS production on programmed cell death in anther tapetum cells and microspore apoptosis [63]. It was revealed that the E3 ubiquitin ligase family SINAR shapes the ideal high-yield panicle of rice through the dual regulation of the receptor-like kinase OsER1 [64]. Light not only provides material energy for plants but also affects the photomorphogenesis of plants. Genes involved in signal transduction, hormone pathways, light responses, and the regulation of organ development were identified to be associated with transgenerational inheritance [65].

## 11. Outlook

With continuously decreasing sequencing costs, genomic and multi-omics studies are expanding across diverse plant species. This trend, combined with advanced gene editing tools and a deeper understanding of plant–environment interactions, is poised to further accelerate the discovery and deployment of genetic solutions for sustainable agriculture in the face of global challenges.
